# Micronutrients attenuate progression of prostate cancer by elevating the endogenous inhibitor of angiogenesis, Platelet Factor-4

**DOI:** 10.1186/1471-2407-10-258

**Published:** 2010-06-04

**Authors:** David Cervi, Brian Pak, Natalie A Venier, Linda M Sugar, Robert K Nam, Neil E Fleshner, Laurence H Klotz, Vasundara Venkateswaran

**Affiliations:** 1 Department of Molecular and Cellular Biology, Sunnybrook Health Sciences Centre, 2075 Bayview Avenue, Toronto, Ontario, M4N 3M5, Canada; 2 Axela Biosensors, 480 University Avenue, Suite 910, Toronto, Ontario, M5G 1V2, Canada; 3 Division of Urology, Sunnybrook Health Sciences Centre, S-118B, 2075 Bayview Avenue, Toronto, Ontario, M4N 3M5, Canada; 4 Department of Pathology, Sunnybrook Health Sciences Centre, E-4, 2075 Bayview Avenue, Toronto, Ontario, M4N 3M5, Canada; 5 Division of Urology, Sunnybrook Health Sciences Centre, MG-408, 2075 Bayview Avenue, Toronto, Ontario, M4N 3M5, Canada; 6 Division of Urology, Princess Margaret Hospital, 3-130, 610 University Avenue, Toronto, Ontario, M5G 2M9, Canada; 7 Department of Surgery, University of Toronto; Division of Urology, Sunnybrook Health Sciences Centre S-118B, 2075 Bayview Avenue, Toronto, Ontario, M4N 3M5, Canada

## Abstract

**Background:**

Longstanding evidence implicates an inadequate diet as a key factor in the onset and progression of prostate cancer. The purpose herein was to discover, validate and characterize functional biomarkers of dietary supplementation capable of suppressing the course of prostate cancer *in vivo*.

**Methods:**

The *Lady *transgenic mouse model that spontaneously develops prostate cancer received a diet supplemented with a micronutrient cocktail of vitamin E, selenium and lycopene ad libitum. A proteomic analysis was conducted to screen for serum biomarkers of this dietary supplementation. Candidate peptides were validated and identified by sequencing and analyzed for their presence within the prostates of all mice by immunohistochemistry.

**Results:**

Dietary supplementation with the combined micronutrients significantly induced the expression of the megakaryocyte-specific inhibitor of angiogenesis, platelet factor-4 (P = 0.0025). This observation was made predominantly in mice lacking tumors and any manifestations associated with progressive disease beyond 37 weeks of life, at which time no survivors remained in the control group (P < 0.0001). While prostates of mice receiving standard chow were enlarged and burdened with poorly differentiated carcinoma, those of mice on the supplemented diet appeared normal. Immunohistochemical analysis revealed marked amplifications of both platelet binding and platelet factor-4 within the blood vessels of prostates from mice receiving micronutrients only.

**Conclusion:**

We present unprecedented data whereby these combined micronutrients effectively promotes tumor dormancy in early prostate cancer, following initiation mutations that may drive the angiogenesis-dependent response of the tumor, by inducing platelet factor-4 expression and concentrating it at the tumor endothelium through enhanced platelet binding.

## Background

Prostate cancer (PCa) remains the second leading cause of cancer related deaths in North American men, although the rate has been declining in part from the use of statin drugs for unrelated medical conditions [1]. In spite of recent reports that place into question the benefit of dietary supplementation with respect to overall survival following diagnosis of PCa, preclinical studies continue to reveal significant benefits using micronutrient cocktails as preventative regimens in spontaneous mouse models of PCa [2].

Among the many micronutrients tested for their anticancer properties, vitamin E, a major intracellular antioxidant, remains one of the most studied [3-8]. We have previously determined that the effect of vitamin E in PCa is mediated, at least in part, by the induction of cell cycle arrest through the modulation of the cdk inhibitor, p27^*Kip1 *^[7]. Selenium has been implicated in playing a chemopreventive role in various cancers, including PCa [8,9]. We have shown that selenium also induces cell cycle arrest *in vitro *but only in the presence of a functional androgen receptor [10]. Finally, the carotenoid, lycopene, has shown considerable clinical benefits in both diabetic and PCa patients, particularly when combined with other tomato nutrients [11]. Lycopene has also been proven to act synergistically as a chemopreventive agent when combined with ketosamines *in vitro *[12]. Aside from the well reported intracellular mechanisms of these micronutrients as single agents, no studies have surfaced to suggest how the host responds to the combination of these micronutrients that may suppress tumor growth. The rationale herein to use all three in combination comes from previous studies conducted in our laboratory [2,13]. We have reported synergistic properties between vitamin E and selenium that induce growth arrest of LNCaP cells *in vitro *over either antioxidant alone [14]. As well, lycopene as a single agent has similarly been shown to be effective *in vitro*, yet this response is not necessarily reflected *in vivo *(unpublished data). Thus, we tested all three in combination for their ability to induce the production of functional biomarkers that could potentially be responsible for the delayed progression of prostate cancer observed in the *Lady *mouse model of PCa [13].

We present here a proteomic approach that has deciphered an anti-angiogenesis response *in vivo *by the combined administration of vitamin E, selenium and lycopene (E/S/L) in a spontaneous mouse model of adenocarcinoma of the prostate. We have found that these micronutrients can induce the expression of PF-4, a megakaryocyte-specific protein that is an endogenous inhibitor of angiogenesis. We propose the mechanism that the subsequent upregulation of PF-4 in platelets upon terminal differentiation of the megakaryocyte allows for the delivery of the elevated protein to the tumor, thereby suppressing tumor-dependant angiogenesis and promoting a dormant phenotype in the *Lady *mouse.

## Methods

### Mice and dietary conditions

Female *Lady *transgenic mice (12T-10) were obtained from Dr. Robert J. Matusik (Vanderbilt Prostate Center, Vanderbilt University Medical Center, Nashville, TN). All animals were housed and maintained according to institutional guidelines set forth by Sunnybrook Health Sciences Centre, Toronto, Canada and in accordance with the Canadian Council on Animal Care. Animal research reported in the manuscript has been performed with the approval of the Sunnybrook Research Ethics Board and conducted as per institutional guidelines set forth by Sunnybrook Health Sciences Centre, Toronto, Canada and in accordance with the Canadian Council on Animal Care. Animals were fed either standard chow or a chow supplemented with the antioxidants, vitamin E (α-tocopherol succinate) (800IU), selenium (seleno-DL methionine) (200 μg) and lycopene (50 mg) (Purina Mills Test Diet, Richmond, Indiana). The latter diet is abbreviated henceforth, E/S/L. All animals were maintained on each diet for a period of 42 weeks.

### Serum processing and Surface-enhanced Laser Desorption Ionization - Time-of-Flight Mass Spectrometry (SELDI-ToF MS)

All mice were anaesthetized by inhalation (Isoflurane-4% induction/2% maintenance) prior to whole blood collection via cardiac puncture using a 1cc syringe fitted with a 26 gauge/0.5 cm needle. The blood was quickly dispensed into 1.5 mL microcentrifuge tubes and allowed to clot on ice. The clotted blood was then centrifuged at 14,000 *g *for 5 minutes followed by collection of the top phase (serum) and storage at -80°C until processing time. Serum (20 μL) was fractionated by anion-exchange chromatography adopted from the Expression Difference Mapping (EDM) Serum Fractionation protocol (Ciphergen, Fremont, CA) in a 96-well format filter plate.

Expression difference mapping (EDM) on ProteinChip arrays was carried out using carboxymethyl ProteinChip arrays (CM10 ProteinChip arrays; Ciphergen, Fremont, CA) and readings were performed using the Protein Biology System IIC (PBSIIC) processed with the ProteinChip Software Biomarker Edition^®^, Version 3.2.0 (Ciphergen, Fremont, CA). After baseline subtraction, spectra were normalized by means of a total ion current. Peak detection was performed by using Biomarker Wizard software (Ciphergen, Fremont, CA) using a minimum peak detection criteria of a signal-to-noise ratio greater than 3.

### Gel purification and sequencing of candidate peptides

A 20 μL sample of the peptide-containing fraction was resolved on NuPAGE^® ^Novex Bis-Tris Gels polyacrylamide gels (Invitrogen) and a SeeBlue^® ^Plus2 Pre-Stained Standard (Invitrogen) was loaded into a single well as reference markers. SilverQuest™ Silver Staining Kit (Invitrogen) was used according to manufacture's protocol to stain peptides after resolving. Extraction of peptides with apparent molecular weights corresponding to those found to be significantly different between the two groups of mice (Table 1) were excised from the gels using a fine (1 mm) pasture pipet. The protein-containing band was in-gel digested with try sin and processed for sequencing according to previously published procedures [15] and subjected to LC-MS/MS analysis (Agilent 1100 HPLC-chip and 6340 ion trap MSD system, Agilent Technologies). Raw MS/MS results were searched against NCBInr restricted to mammals' subset database, using Spectrum Mill MS Proteomics Workbench (Agilent Technologies). Proteins with two or more peptides identified from MS/MS search were reported.

**Table 1:** Candidate serum biomarkers of E/S/L-supplementation.

			Average Peak Intensity	
			
Fraction (pH)	Peak Mass (Da)	*P*	Standard Diet	Supplemented Diet
9.0	4479.11	0.0019	0.424	0.624
9.0	7489.44	0.0041	0.379	0.224
7.0	6267.37	0.0065	2.105	3.963
7.0	6483.69	0.0012	0.176	0.481
7.0	6824.78	0.0065	2.805	5.143
7.0*	8963.14	0.0025	0.239	0.531
4.0	2297.16	0.0065	2.566	1.182
4.0	2927.47	0.0065	1.090	0.571
4.0	5586.30	0.0019	1.163	0.659
3.0	2902.33	0.0055	0.585	0.240

*PF-4

Serum obtained at the end of the study period from *Lady *mice receiving a standard diet alone or an E/S/L-supplemented diet were subjected to a standard biomarker discovery protocol according to Materials and Methods. Ten peptides in total were observed to be significantly deregulated with respect to their serum concentrations (Table 1, p < 0.01). Molecular weights of these peptides ranged between 2297 and 8963 Da with the majority of proteins exhibiting alkaline properties (high isoelectric point (pI)) based on their binding properties to the chromatographic resins used in their discovery.

### Tissue sampling and immunohistochemistry

All animals were examined for gross abnormalities according to previously described methods [2,13]. Prostate glands were fixed in 10% (v/v) buffered formalin, paraffin-embedded and cut into 5 μm sections mounted onto glass slides. The sections were deparaffinized with xylene, rehydrated and boiled for 10 min in citrate buffer (pH 7.0). They were then blocked with 0.3% hydrogen peroxide in methanol followed by normal serum and then incubated overnight at 4°C with the primary antibody against mouse PF-4 (anti-PF-4, mouse monoclonal antibody (Santa-Cruz Biotechnology) diluted 1:70 in PBS) or against anti-CD41 (anti-CD41, rat monoclonal antibody (Abcam) diluted 1:50 in PBS). Slides were then reacted with biotin-labeled anti-mouse IgG/anti-rat IgG and incubated with preformed avidin-biotin peroxidase complex (Vector Laboratories). Sections were counterstained with hematoxylin, dehydrated, and mounted. The expressions of both PF-4 and CD41 were scored based on the intensity of staining (absence of staining, weak or strong) by two independent investigators.

## Results

### Reduced tumor burden and extended survival are observed for mice receiving a supplemented diet of E/S/L

*Lady *transgenic mice were placed on a standard diet or a diet supplemented with vitamin E (800 IU), selenium (200 μg) and lycopene (50 mg) for a period of 37 weeks from weaning. We have previously shown that by this time, 90% of animals on a standard diet develop prostate cancer, but that 85% of animals administered the micronutrients have completely normal, benign prostates. Similar gross and histological differences between the two groups were observed here as with our previous study, with an overall significant survival benefit achieved for mice consuming the supplemented diet [2,13].

### Serum proteomic analysis reveals an upregulation of PF-4 in *Lady *transgenic mice receiving E/S/L-supplemented diets

**Figure 1:**
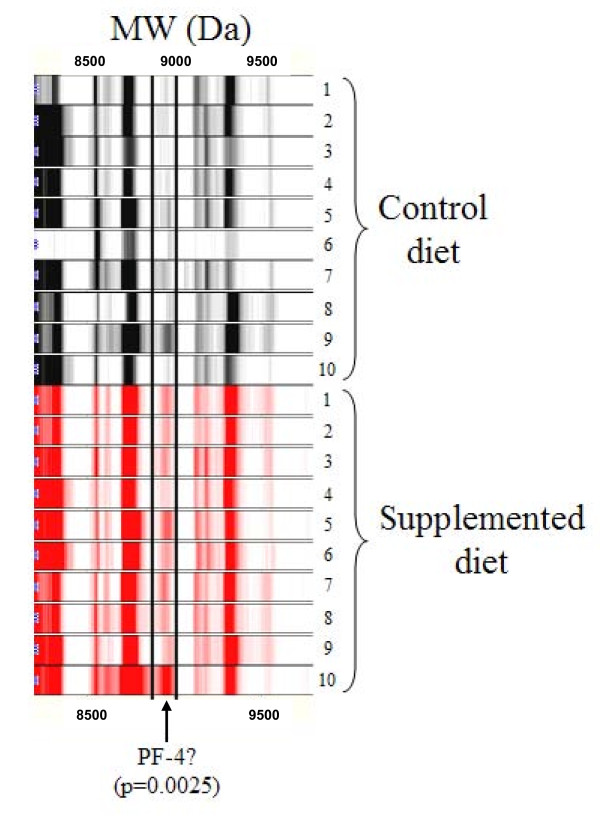
**Validation and identification of serum PF-4**. Serum was harvested from *Lady *mice receiving either a standard diet (black) (n = 10) or a E/S/L-supplemented diet (red) (n = 10) at the study endpoint (37 weeks). A standard comparison was made using the SELDI-ToF biomarker discovery method. A spectral readout from SELDI-ToF MS between the two groups is presented here in gel-view format with a specific emphasis on the banding pattern related to the peptide candidate with an apparent molecular weight of 8960 Da.

**Figure 2:**
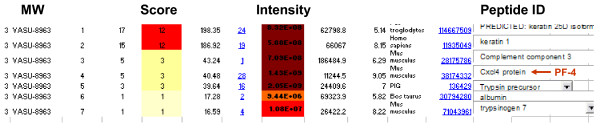
**Peptide purification and sequencing**. Intensity differences of the bands are reflective of the serum concentrations of the peptide in each mouse. This peptide was later purified and sequenced according to Materials and Methods and identified as murine CXCL4 (PF-4). Contaminating peptides from serum processing and handling are shown as well in the sequencing readout.

Serum obtained at the end of the study period from *Lady *mice receiving a standard diet alone or an E/S/L-supplemented diet were subjected to a standard biomarker discovery protocol according to Materials and Methods. Ten peptides in total were observed to be significantly deregulated with respect to their serum concentrations (Table 1, p < 0.01). Molecular weights of these peptides ranged between 2297 and 8963 Da with the majority of proteins exhibiting alkaline properties (high isoelectric point (pI)) based on their binding properties to the chromatographic resins used in their discovery. Following purification of the fractionated specimens outlined in Table 1 for sequencing purposes, the resolving power of gel electrophoresis limited the number of candidates for sequencing to a single, prominent band with an apparent molecular weight approximating 9000 Da (data not shown); corresponding to the 8963 Da candidate biomarker discovered by SELDI-ToF (Table 1). The expression profiles obtained for this candidate biomarker during the initial discovery phase revealed it to be significantly upregulated in the majority of *Lady *mice receiving a E/S/L-supplemented diet (Fig 1, p = 0.0025). Analysis by MS/MS of the 8963 Da peptide showed a high confidence match with murine chemokine (C-X-C motif) ligand 4 (CXCL4), henceforth referred to here as, PF-4 (Fig 2).

### Amplified intravascular PF-4 and platelet binding in prostates of *Lady *mice administered E/S/L-supplemented diets

**Figure 3:**
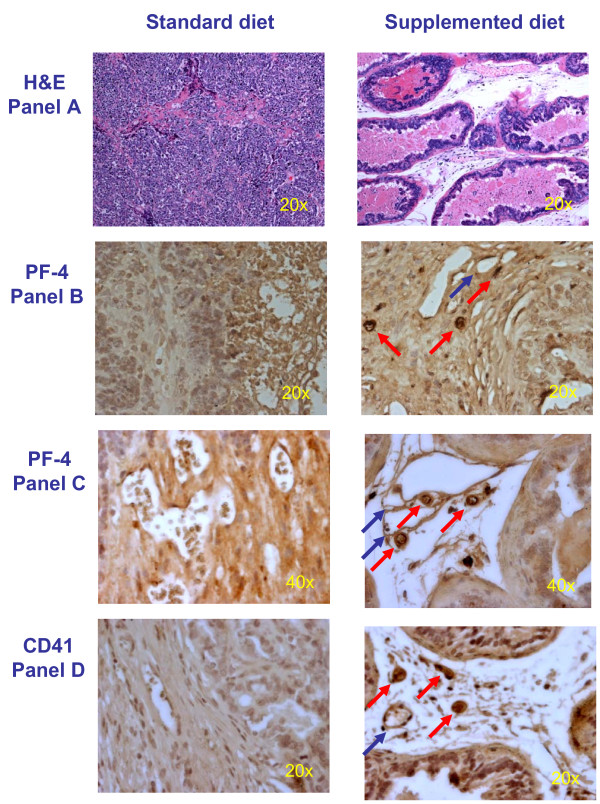
**Histopathology and immunohistochemistry of platelet binding and PF-4 in prostates of the *Lady *mouse**. Prostate glands from *Lady *mice receiving either standard diet or an E/S/L-supplemented diet were excised and processed for histomorphology and immunohistochemistry according to Materials and Methods. H&E staining reveals tumor burden with poorly differentiated carcinoma in prostates of mice receiving a standard diet, while a normal prostate architecture is evident in mice receiving an E/S/L-supplemented diet **(Panel A, 20× magnification)**. Distinct intravascular staining for PF-4 is evident in prostates of *Lady *mice receiving E/S/L compared to that of the control group **(red arrows, Panel B-20X and Panel C-40X magnification, respectively)**. No positive staining for PF-4 is evident in the lymphatics **(blue arrows)**. Distinct intravascular staining for platelets is evident in prostates of *Lady *mice receiving the test diet compared to that of the control group **(red arrows, Panel D-20X magnification)**. No platelet binding is evident in the lymphatics **(blue arrows)**.

To ascertain the proteomic analysis and validation of elevated PF-4 in *Lady *mice receiving an E/S/L-supplemented diet, prostate tissues were processed and immunostained for PF-4 and α_2β_-integrin (CD41), the latter being a surface marker of platelets, which are the biological vehicles of PF-4 *in vivo *(see discussion). Histopathology of prostates was first assessed by H&E for all animals in both groups. While prostates from mice on a standard diet were grossly enlarged and burdened with poorly differentiated carcinoma (74%), those from mice receiving an E/S/L-supplemented diet were normal, both grossly and histologically [2,13]. A representative field is viewed in Fig 3; Panel A. Analysis of PF-4 staining between the two groups revealed intense staining of the protein within the vasculature of the prostate in mice receiving an E/S/L-supplemented diet, but not within the lymphatic ducts (7 out of 8 or 88%). However, along with a complete loss of tissue integrity no discernable staining for PF-4 was observed in the prostates of control mice (1 out of 7 had a very weak staining or 14%) (Fig 3; Panel B-20X magnification, Panel C-40X). Finally, given the negative staining for PF-4 in the prostate epithelium and connective tissues and the fact that this protein has solely been shown to be megakaryocyte-specific, we sought to confirm its elevated presence in the vasculature of the prostate to be attributed to the increased binding capacity of circulating platelets. As depicted in Fig 3; Panel D, the intensity differences in CD41 staining; i.e., platelet binding, between the two groups are akin to those observed for PF-4. The vast majority of mice receiving a E/S/L-supplemented diet therefore exhibited a greater number of PF-4-containing platelets bound to the blood vessels of the prostate (5 out of 6 or 83%) versus none for those in the control group (0 out of 6).

## Discussion

The micronutrients, vitamin E, selenium and lycopene are known to provide cells with an incredibly efficient free-radical scavenging system, therefore substantially reducing cancer-promoting DNA damage. While the literature evidence and current understanding of each supplement strongly supports this view, we report an unprecedented finding here that the combination of these three supplements together, but not individually, is also antiangiogenic by virtue of its ability to induce the expression of a megakaryocyte-specific, endogenous inhibitor of angiogenesis, PF-4. Furthermore, E/S/L has the ability to promote platelet binding to an activated endothelium and therefore concentrate this inhibitor at susceptible sites; i.e., tumor-adjacent vasculature.

The initial goal of this study was to conduct a full serum proteomic analysis to identify and characterize functional biomarkers of antioxidant supplementation likely to have an impact on the course of prostate cancer in a spontaneous PCa mouse model. We have previously reported an extended survival benefit in the *Lady *transgenic mouse supplemented with an E/S/L cocktail and therefore wanted to further investigate the molecular mechanisms *in vivo *governed by their synergistic properties that could ultimately be capable of maintaining the extrinsically mutated prostate epithelium in check [2,13]. Individually, these antioxidants imparted no benefits compared to those observed in the combination group; i.e., reduced tumor burden and extended survival [2]. Serum profiling was therefore restricted to compare *Lady *transgenic mice receiving a standard diet with those receiving E/S/L-supplementation, as the latter group alone clearly exhibited the benefits. It is important to note that while we have successfully identified PF-4 as a biomarker of dietary response by differential expression, validation and isolation, univariate data analysis of the complete proteome validated an additional 9, differentially expressed peptides (p < 0.01) between these two groups of mice (Table 1). These peptides ranged in molecular weights of 2200-7400 Da. Their identifications, however, have been hampered largely by inadequate resolving power of gel electrophoresis. It is conceivable, therefore, that the expression patterns of other proteins, in addition to PF-4, may be playing as intricate a role in controlling the progression of early-stage prostate cancer.

Having identified PF-4 as a dietary biomarker of E/S/L-supplementation implicates a very intricate biologic pathway that alters the hemostatic balance *in vivo *to subsequently suppress the activated endothelium in the cancerous prostate. This response likely lies initially at the level of the megakaryocyte since it is the sole source for PF-4 that is currently known *in vivo *[16-18]. In fact, our findings here indicate that the prostate gland stained negatively for PF-4 and that the intense staining pattern was restricted to the intravascular regions of the gland, but only in mice receiving the E/S/L cocktail. In light of a recent report suggesting that upregulation of PF-4 may play a crucial role in the early stages of several cancers [19], we propose here that upregulation of this peptide in our study was crucial in preventing angiogenesis at the tumor site in early prostate cancer. More importantly, this response could have only been initially mediated via a biologic modulation of the megakaryocyte located at a distant site from the tumor itself (bone marrow and spleen). Platelets, being the endpoint of megakaryocyte differentiation, now carrying an increased load of PF-4, would later bind to the activated endothelium of the cancerous prostate where it would subsequently suppress the angiogenic drive induced by the developing tumor following its angiogenic switch. The existence of such a switch has been long proposed and recently proven in tumor biology [20,21]. Overall, our findings of elevated PF-4 and improved shuttling of the protein suggest that *in vivo*, such properties can preclude the angiogenic drive of most tumors, particularly following initiation mutations governing the angiogenic switch. In doing so, the tumor can be kept in check by the host during its earliest stages and prevented from further localized invasion and metastatic spread, albeit in the presence of E/S/L. This is the preclinical interpretation that we propose in light of our findings herein and the current literature pertaining to tumor angiogenesis.

We have considered an additional mechanism to our observed benefit of E/S/L-supplementation in the *Lady *mouse. Platelet activation and binding, which are intrinsic functions of platelets and occur irrespective of dietary supplementation, should have occurred in both groups of animals, which indeed was the case. However, mice receiving the cocktail exhibited a greater degree of platelet binding capacity (↑α_2β_-integrin) to the activated endothelium of the cancerous prostate. Although any mechanism suggested at this stage would be purely speculative we have considered that differences in platelet numbers alone between the two groups (reduced in tumor-bearing animals versus elevated in treated animals) may have perhaps contributed to such apparent differences. However, for E/S/L-supplementation to promote platelet production it would necessitate having to increase the number of its megakaryocyte precursors in the hematopoietic compartments of *Lady *mice. Analysis of spleens obtained from both groups indicated no such differences in the current study (data not shown). Furthermore, there has been no indication in our studies that changes in platelet numbers results from progressive disease or to dietary supplementation (unpublished results).

## Conclusions

The findings we are reporting here outline a completely novel biologic mechanism involving the use of well documented micronutrients as a preventative therapy for the progression of prostate cancer. We have provided unprecedented data to prove that this combined supplementation is a *natural *antiangiogenic therapy by virtue of its ability to upregulate an endogenous inhibitor of angiogenesis, PF-4 (p = 0.0025). While a family of micronutrients, referred to in the literature as neutriceuticals, have shown to be antiangiogenic, these studies have supported such a role through altered signalling pathways related to oxidative stress in endothelial cells [22,23]. We, however, have serendipitously discovered this particular antioxidant cocktail to be antiangiogenic by an indirect physiological pathway involving the platelet precursor, the megakaryocyte. Implicit in this finding is the potential clinical benefit that can be achieved through dietary supplementation, particularly during the earliest stages of cancer development and at a time when they become angiogenesis-dependant.

## Competing interests

The authors declare that they have no competing interests.

## Authors' contributions

DC was involved in conducting the experiments, BP designed and conducting experiments, NAV assisted with the experiments, LMS assisted in the pathology and interpretation of results, RKN provided intellectual input, NEF assisted in the design and intellectual input as well as in the statistical component, LHK helped with the design and intellectual input as well as data interpretation, VV coordinated the study in terms of design, interpretation, intellectual contribution and manuscript drafting.

All authors have read and approved the final manuscript.

## Funding

Grant Funding to VV from CPCRI and Deans Fund.

## Pre-publication history

The pre-publication history for this paper can be accessed here:

http://www.biomedcentral.com/1471-2407/10/258/prepub
